# Association between IL-1β + 3954C/T polymorphism and myocardial infarction risk: A meta-analysis

**DOI:** 10.1097/MD.0000000000011645

**Published:** 2018-07-27

**Authors:** Yizhen Fang, Huabin Xie, Zhiyuan Lin

**Affiliations:** a Xiamen University Affiliated Cardiovascular Hospital; b Center for Clinical Laboratory, Xiamen University Affiliated Zhongshan Hospital, Xiamen, Fujian, China.

**Keywords:** IL-1β, meta-analysis, myocardial infarction, polymorphism

## Abstract

**Background:**

Many studies have reported that the IL-1β + 3954C/T polymorphism (rs1143634) is related to myocardial infarction (MI). To classify the association between IL-1β + 3954C/T and MI susceptibility, we performed a meta-analysis.

**Methods:**

We retrieved relevant literature from electronic databases (Embase, PubMed, Cochrane, and Web of Science). Pooled odds ratios (ORs) and 95% confidence intervals (95% CIs) were calculated with a fixed effect model or a random effect model. Sensitivity analysis and publication bias results are also presented.

**Results:**

Nine eligible studies (2299 controls and 2203 cases) were included. The pooled results showed a significant relationship between MI and IL-1β + 3954C/T in an allelic comparison (T vs C: OR = 1.13, 95% CI 1.02–1.25, *I*^2^ = 0%, *P*_H_ = .448) and in a dominant model (TC + TT vs CC: OR = 1.15, 95% CI 1.02–1.30, *I*^2^ = 0%, *P*_H_ = .880). Ethnic subgroup analysis showed similar results in Caucasian populations: an allelic comparison (T vs C: OR = 1.16, 95% CI 1.04–1.29, *I*^2^ = 0%, *P*_H_ = .701), homozygote model (TT vs CC: OR = 1.36, 95% CI 1.04–1.79, *I*^2^ = 0%, *P*_H_ = .673), and dominant model (TC + TT vs CC: OR = 1.17, 95% CI 1.02–1.33, *I*^2^ = 0%, *P*_H_ = .851). In addition, similar effects remained in subgroups analyses of high-quality studies and PCR-RFLP (restriction fragment length polymorphism) data.

**Conclusion:**

Our meta-analysis proved that IL-1β + 3954C/T is associated with MI susceptibility, especially among Caucasian populations.

## 1 Introduction

Myocardial infarction (MI), a highly prevalent cardiac emergency caused by a vital disequilibrium between oxygen supply and demand in myocardial cells, is a primary cause of morbidity and mortality worldwide.^[1]^ MI is an incredibly complex disease. Most MIs are related to coronary artery disease (CAD) and coronary atherosclerosis rupture is the most frequent cause of MI.^[2,3]^ It is well known that inflammation plays a primary role in atherosclerosis^[4]^ and plaque rupture.^[5]^ Inflammation seems to influence each stage of atherosclerotic development, such as oxidative injury,^[6]^ cell proliferation, and plaque evolution and instability.^[7,8]^

The pro-inflammatory cytokine interleukin-1 beta (IL-1β) is involved in the initiation of multiple biological cascades that are important parts of the inflammatory reaction.^[9]^ IL-1β plays a key role in coronary atherosclerotic heart disease^[10]^ and atherosclerotic inflammation.^[11]^ Some studies have reported that the expression of IL-1β was elevated in the myocardium early after injury.^[12,13]^ A single nucleotide polymorphism (SNP) was identified in exon 5 at position +3954C/T of the *IL-1β* gene. The T allele of IL-1β + 3954C/T is less common than the С allele and is related to an elevated serum IL-1β level.^[14]^ A polymorphism leading to IL-1β overproduction may increase the risk of autoimmune diseases such as atherosclerosis.^[14]^ Although many studies have presented a connection between IL-1β + 3954C/T and MI risk,^[15–23]^ the sample sizes of these studies were limited, and the results are controversial. Thus, we performed a meta-analysis to clarify the association between IL-1β + 3954C/T and MI susceptibility.

## 2 Materials and methods

### 2.1 Search strategy

A systematic search was performed in PubMed, Cochrane, Embase (Excerpta Medica Database), and Web of Science. The systematic search included articles published up to November 30, 2017. The following search terms were combined: “(SNP or SNPs or “single nucleotide polymorphism” or polymorphism or “genetic polymorphism” or mutation or variant or variation),” “(“heart infarction” or “myocardial infarction” or MI or “myocardial infarct” or “ischemic heart disease” or “acute coronary syndrome” or “coronary artery disease”),“ and “(IL-1β or ” interleukin-1 beta” or “IL-1 beta” or IL-1B).” Language and publication year were not restricted in our search. Finally, 1353 articles were retrieved using the aforementioned terms.

### 2.2 Inclusion and exclusion criteria

Eligible articles conformed to the following inclusion criteria: assessed MI as the outcome of study; assessed the association between MI and IL-1β + 3954C/T (rs1143634); presented genotype data of cases and controls with risk of MI sufficient to calculate odds ratios (ORs) and 95% confidence interval (CIs); and used a case–control design for human. Exclusion criteria included deficient genotype frequency; duplicate literature; published as a letter, comment, or review; evaluated other IL-1β SNPs and not rs1143634; case-only study; and not a human study. Two investigators separately selected the potential literature according to these criteria. When divergences appeared, the third investigator made the final decision.

### 2.3 Data extraction

Information from all eligible literature was extracted by 2 authors independently. The third author handled any divergences until agreement among all authors was unanimous. The following data were collected: name of first author, ethnicity of subjects, Hardy–Winberg equilibrium (HWE), sample size, genotyping method, genotype distributions in cases and controls, and the quality of study. Ethnicity was classified as Asian or Caucasian. We sent requests to corresponding authors for additional data when the primary data could not be obtained from relevant articles.

### 2.4 Quality score assessment

The quality of eligible literature was accessed by 2 authors separately according to predetermined criteria (Table 1), which were adjusted and revised from previous articles^[24,25]^ and the Newcastle–Ottawa Scale (NOS). The adjusted criteria contained many items, such as the source of controls, the source of cases, case–control matching, sample size, genotyping method, and the HWE in controls. Two authors separately graded all included studies and any divergence was assessed by the third author. Scores ranged from 0 to 10. A study quality score ≥6 indicated “high quality,” while a study quality score <6 indicated “low quality.”^[26]^

**Table 1:**
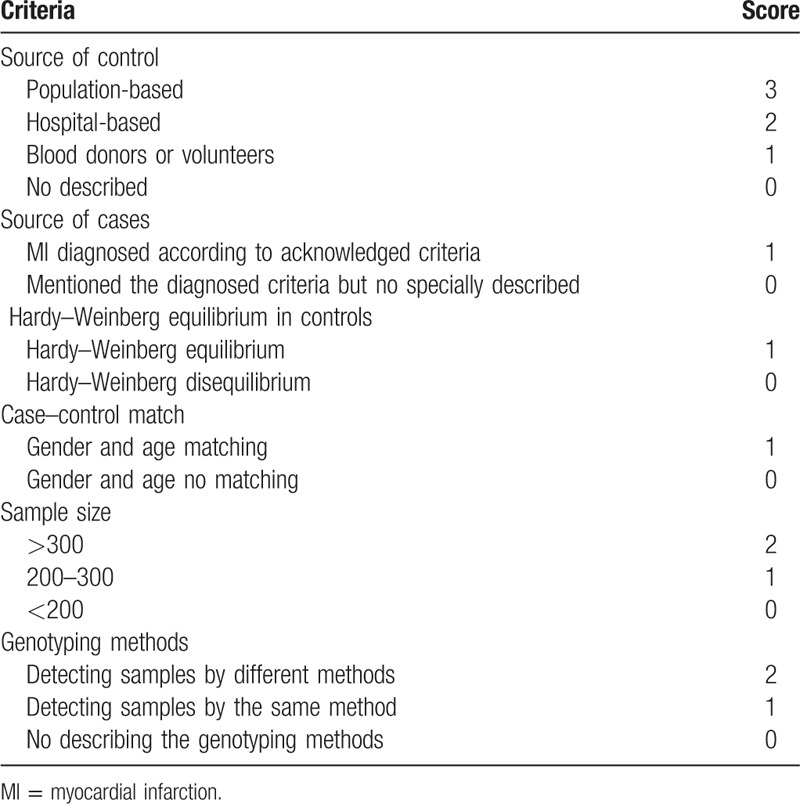
Quality evaluation tabulation.

### 2.5 Statistical methods

The meta-analysis was performed according to the PRISMA checklist and followed these guidelines.^[27]^ The control group in each included study was assessed for HWE by a Chi-square test, and a group was considered to be in Hardy–Weinberg disequilibrium at *P* < .05. ORs and 95% CIs were calculated to assess the strength of the association between IL-1β + 3954C/T and MI risk. The pooled ORs were used to assess allelic comparison (T vs C), a heterozygote model (TC vs CC), a homozygote model (TT vs CC), a dominant model (TT + TC vs CC), and a recessive model (TT vs TC + CC). Heterogeneity was assessed by the Q statistic (significant value at *P* < .1) and the *I*^2^ statistic (*I*^2^ > 50% indicating a significant inconsistency).^[28]^ When heterogeneity existed, we carried out a random effect model (the DerSimonian and Laird method) to evaluate the pooled ORs and 95% CIs, otherwise, a fixed effect model (Mantel–Haenszel method) was performed to assess the pooled ORs and 95% CIs. Sensitivity analysis was performed by examining the effect of omitting individual studies. Begg funnel plot and Egger test were carried out to check for the publication bias (*P* < .05 suggested a significant bias). STATA software (version 12.0; StataCorp, College Station, TX) was used to perform all the tests in our meta-analysis, with 2-sided *P* values.

All analyses were based on previous published studies; thus, no ethical approval and patient consent are required.

## 3 Results

### 3.1 Characteristics of studies

**Figure 1:**
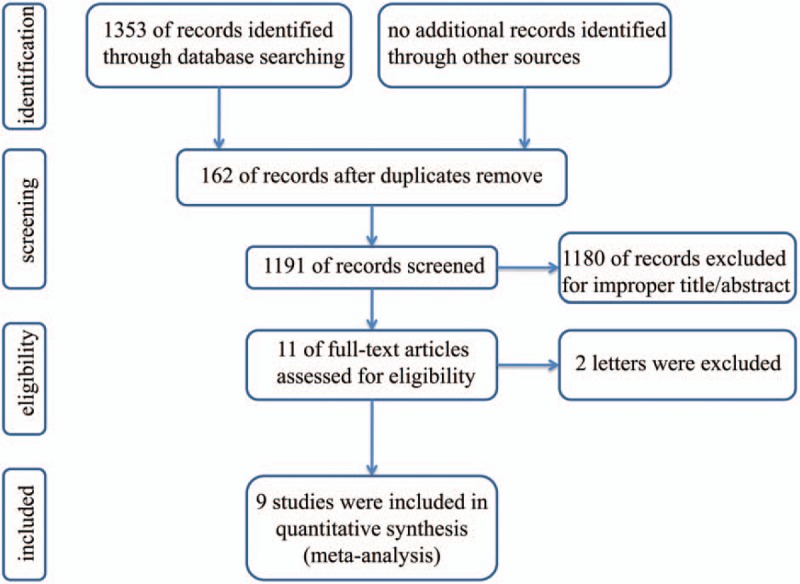
Flow chart of study selection.

A total of 1353 studies were identified from the PubMed, Cochrane, Embase, and Web of Science databases. The flow diagram in Fig. 1 shows the literature screening process. We excluded 1342 articles, including 162 articles presenting repeated findings and 1180 irrelevant articles. The remaining 11 articles were full-text articles. Then, 2 letters^[29,30]^ were excluded. Eventually, 9 eligible case–control publications, all conforming to the inclusion criteria, were included in our meta-analysis.

The 9 independent studies included in our meta-analysis included 2203 cases and 2299 controls.^[15–23]^Table 2 summarizes the main features of each study. Two studies were based on Asian populations,^[16,19]^ while the other studies were based on Caucasian populations.^[15,17,18,20–23]^ The results of the HWE tests for genotypic distribution in controls are summarized in Table 2. Quality scores for included articles ranged from 4 to 8, with 78% (7 of 9) of the studies being of high quality (score ≥6).

**Table 2:**
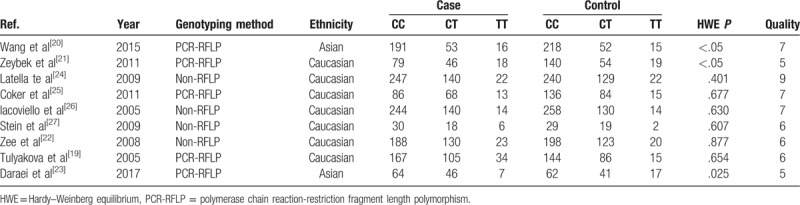
Characteristics of studies included in the meta-analysis.

### 3.2 Meta-analysis results

**Figure 2:**
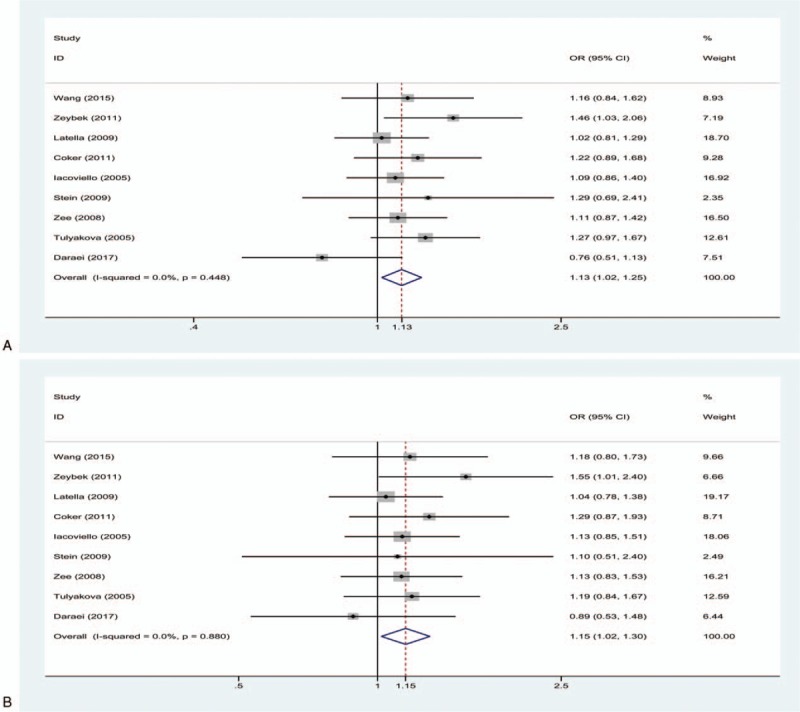
(A) Forest plot for the allelic comparison of IL-1β + 3954C/T in the overall comparison (T vs C), fixed effect model; (B) Forest plot for the dominant model of IL-1β + 3954C/T in the overall comparison (TC + TT vs CC), fixed effect model. The size of the black squares represents the weight of the study in the meta-analysis. The rhombus represents the combined OR. OR = odds ratio.

**Figure 3:**
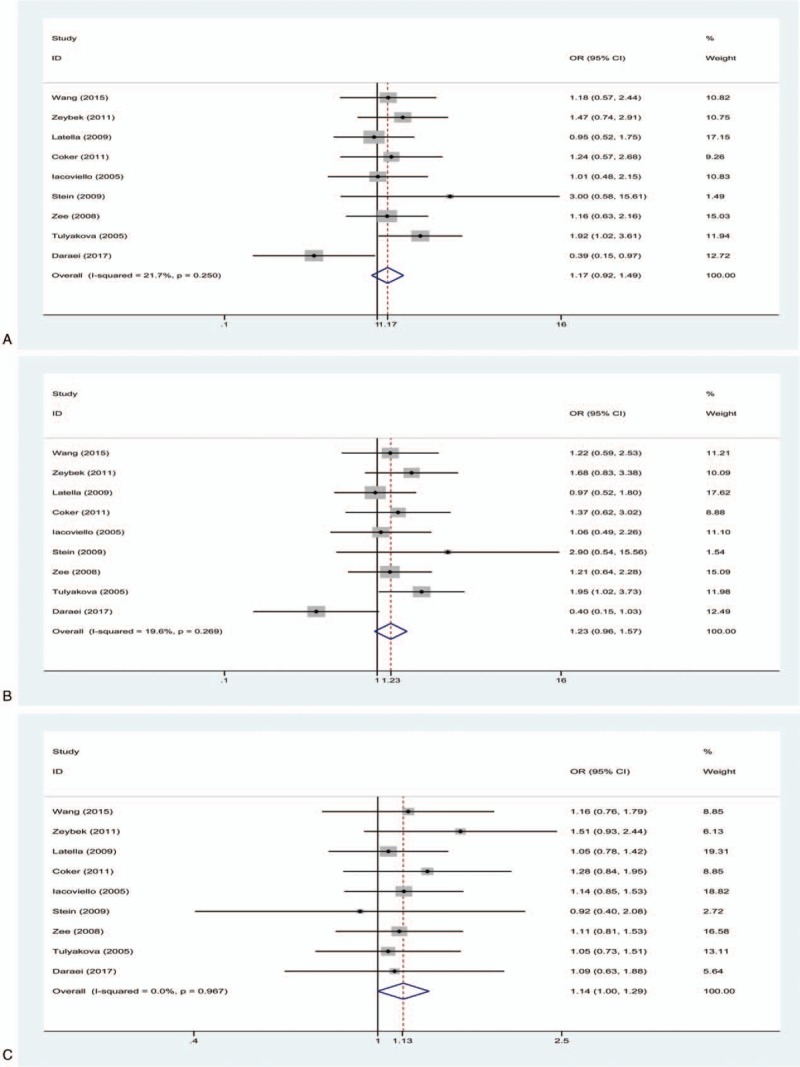
(A) Forest plot for the recessive model of IL-1β + 3954C/T in the overall comparison (TT vs TC + CC), fixed effect model; (B) Forest plot for the homozygote model in the overall comparison (TT vs CC), fixed effect model; (C) Forest plot for the heterozygote model of IL-1β + 3954C/T in the overall comparison (TC vs CC), fixed effect model. The size of the black square represents the weight of the study in the meta-analysis. The rhombus represents the combined OR. OR = odds ratio.

The pooled results showed that a significantly increased risk of MI susceptibility was observed in the allelic comparison (T vs C: OR = 1.13, 95% CI 1.02–1.25, *I*^2^ = 0%, *P*_H_ = .448) and dominant model (TC + TT vs CC: OR = 1.15, 95% CI 1.02–1.30, *I*^2^ = 0%, *P*_H_ = .880) (Fig. 2). No statistically significant association between MI susceptibility and IL-1β + 3954C/T was found in the recessive model (TT vs TC + CC: OR = 1.17, 95% CI 0.92–1.49, *I*^2^ = 21.7%, *P*_H_ = .250), homozygote model (TT vs CC: OR = 1.23, 95% CI 0.96–1.57, *I*^2^ = 19.6%, *P*_H_ = .269), or heterozygote model (TC vs CC: OR = 1.14, 95% CI 1.00–1.29, *I*^2^ = 0%, *P*_H_ = .967) (Fig. 3).

### 3.3 Subgroup analysis

Subgroup analysis by ethnicity showed similar effects in Caucasian populations. There was a significant risk of MI susceptibility in the allelic comparison (T vs C: OR = 1.16, 95% CI 1.04–1.29, *I*^2^ = 0%, *P*_H_ = .701), homozygote model (TT vs CC: OR = 1.36, 95% CI 1.04–1.79, *I*^2^ = 0%, *P*_H_ = .673), and dominant model (TC + TT vs CC: OR = 1.17, 95% CI 1.02–1.33, *I*^2^ = 0%, *P*_H_ = .851). Nevertheless, no significant association was observed in the recessive model (TT vs TC + CC: OR = 1.30, 95% CI 1.00–1.70, *I*^2^ = 0%, *P*_H_ = .659) or heterozygote model (TC vs CC: OR = 1.14, 95% CI 0.99–1.30, *I*^2^ = 0%, *P*_H_ = .885) (Table 3). However, no significant results were found in Asian populations (T vs C: OR = 0.98, 95% CI 0.76–1.26, *I*^2^ = 61.6%, *P*_H_ = .107; TC vs CC: OR = 1.13, 95% CI 0.81–1.59, *I*^2^ = 0%, *P*_H_ = .848; TT vs CC: OR = 0.79, 95% CI 0.45–1.38, *I*^2^ = 70.1%, *P*_H_ = .067; TC + TT vs CC: OR = 1.06, 95% CI 0.78–1.44, *I*^2^ = 0%, *P*_H_ = .386; TT vs TC + CC: OR = 0.75, 95% CI 0.43–1.31, *I*^2^ = 71.5%, *P*_H_ = .061) (Table 3).

**Table 3:**
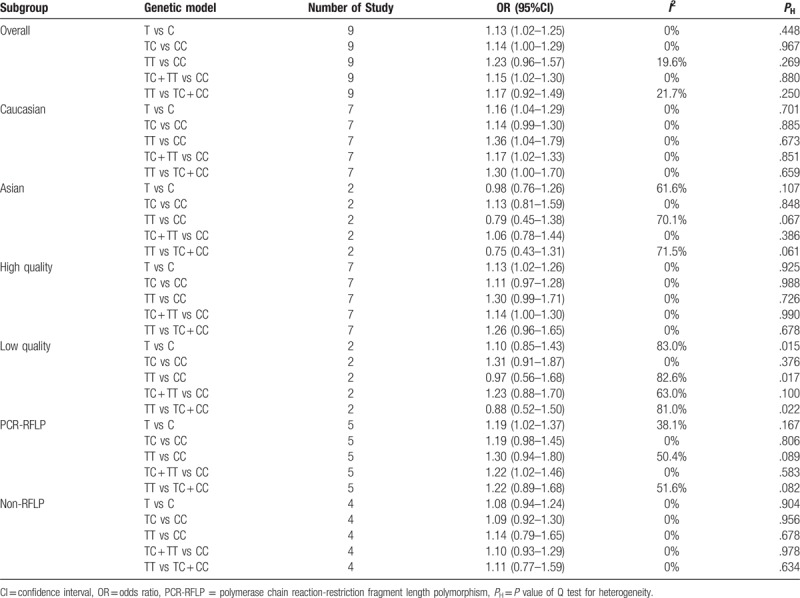
Summary of polled odds ratios in the meta-analysis.

Then, we performed another subgroup analysis to investigate the effect of study quality. Among the high-quality studies, there was a positive association in the allelic comparison (T vs C: OR = 1.13, 95% CI 1.02–1.26, *I*^2^ = 0%, *P*_H_ = .925), but there was no evidence of a significant link in the other genetic models (TC vs CC: OR = 1.11, 95% CI 0.97–1.28, *I*^2^ = 0%, *P*_H_ = .988; TT vs CC: OR = 1.30, 95% CI 0.99–1.71, *I*^2^ = 0%, *P*_H_ = .726; TC + TT vs CC: OR = 1.14, 95% CI 1.00–1.30, *I*^2^ = 0%, *P*_H_ = .990; TT vs TC + CC: OR = 1.26, 95% CI 0.96–1.65, *I*^2^ = 0%, *P*_H_ = .678). No significant effects were observed in the low-quality studies (T vs C: OR = 1.10, 95% CI 0.85–1.43, *I*^2^ = 83.0%, *P*_H_ = .015; TT vs CC: OR = 0.97, 95% CI 0.56–1.68, *I*^2^ = 82.6%, *P*_H_ = .017; TT vs TC + CC: OR = 0.88, 95% CI 0.52–1.50, *I*^2^ = 81.0%, *P*_H_ = .022; TC + TT vs CC: OR = 1.23, 95% CI 0.88–1.70, *I*^2^ = 63.0%, *P*_H_ = .100; TC vs CC: OR = 1.31, 95% CI 0.91–1.87, *I*^2^ = 0%, *P*_H_ = .376) (Table 3).

When stratifying findings by genotyping method, several significant results were detected in the PCR-RFLP subgroup (T vs C: OR = 1.19, 95% CI 1.02–1.37, *I*^2^ = 38.1%, *P*_H_ = .167; TC + TT vs CC: OR = 1.22, 95% CI 1.02–1.46, *I*^2^ = 0%, *P*_H_ = .583), but there was no statistically significant association in the heterozygote model, homozygote model, or recessive model (TC vs CC: OR = 1.19, 95% CI 0.98–1.45, *I*^2^ = 0%, *P*_H_ = .806; TT vs CC: OR = 1.30, 95% CI 0.94–1.80, *I*^2^ = 50.4%, *P*_H_ = .089; TT vs TC + CC: OR = 1.22, 95% CI 0.89–1.68, *I*^2^ = 51.6%, *P*_H_ = .082). No significant association was observed in the non-RFLP subgroup (Table 3).

### 3.4 Sensitivity analysis

**Figure 4:**
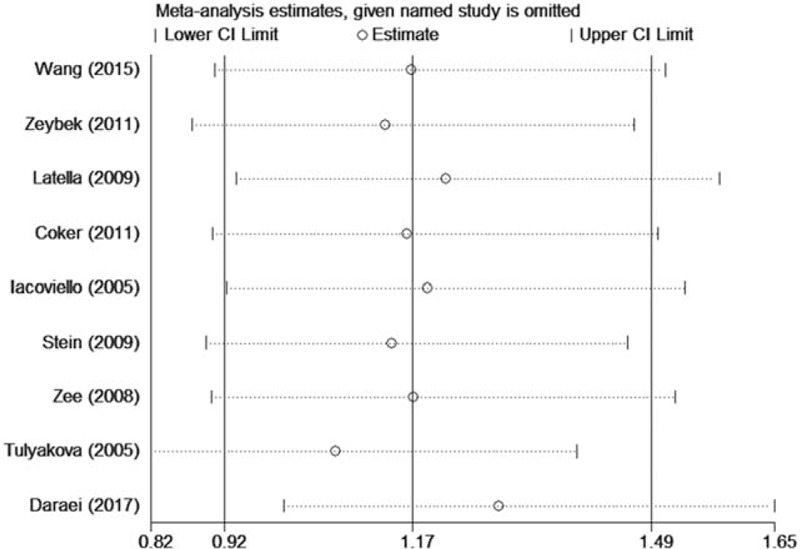
Sensitivity analysis for IL-1β + 3954C/T in the recessive model (TT vs TC + CC).

We detected the effect of individual studies on the pooled OR for IL-1β + 3954C/T by sensitivity analysis. Consistently, the pooled estimate showed no significant change when any single study was omitted, one at a time, from each meta-analysis (Fig. 4).

### 3.5 Publication bias

**Figure 5:**
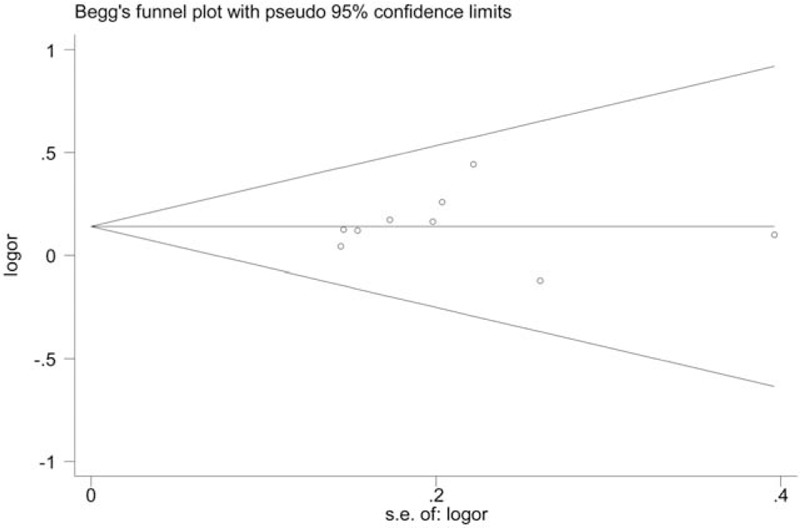
Begg funnel plot showing the publication bias analysis for IL-1β + 3954C/T (TC + TT vs CC).

We evaluated the publication bias by Begg funnel plot and Egger test. The results indicated no significant publication bias in any of the genetic models. Figure 5 shows Begg funnel plot in the allelic comparison (TC + TT vs CC, *P* = .767). Information concerning Egger test are listed in Table 4.

**Table 4:**
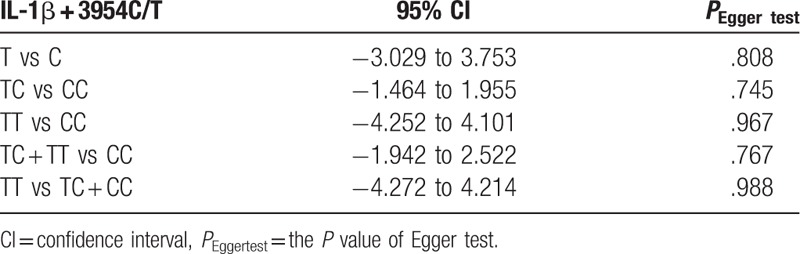
Egger test results.

## 4 Discussion

In our meta-analysis, 9 eligible studies,^[15–23]^ including 2203 cases and 2299 controls, were identified and analyzed. The pooled results showed that IL-1β + 3954C/T significantly increased MI susceptibility in the allelic comparison (T vs C: OR = 1.13, 95% CI 1.02–1.25, *I*^2^ = 0%, *P*_H_ = .448) and the dominant model (TC + TT vs CC: OR = 1.15, 95% CI 1.02–1.30, *I*^2^ = 0%, *P*_H_ = .880). Similar results were observed in Caucasian populations (T vs C: OR = 1.16, 95% CI 1.04–1.29, *I*^2^ = 0%, *P*_H_ = .701; TC + TT vs CC: OR = 1.17, 95% CI 1.02–1.33, *I*^2^ = 0%, *P*_H_ = .851; TT vs CC: OR = 1.36, 95% CI 1.04–1.79, *I*^2^ = 0%, *P*_H_ = .673), and in subgroups from high-quality studies (T vs C: OR = 1.13, 95% CI 1.02–1.26, *I*^2^ = 0%, *P*_H_ = .925) and PCR-RFLP (T vs C: OR = 1.19, 95% CI 1.02–1.37, *I*^2^ = 38.1%, *P*_H_ = .167; TC + TT vs CC: OR = 1.22, 95% CI 1.02–1.46, *I*^2^ = 0%, *P*_H_ = .583). Heterogeneity was not observed in any of the 5 genetic models used.

In the subgroup analysis according to the quality of the studies and genotyping method, the results for the PCR-RFLP subgroup were consistent with the pooled results, and the results for the high-quality study subgroup remained similar in the allelic comparison. However, for the low-quality studies and non-RFLP subgroup, different results were observed in all genetic models. These differences may be due to the smaller sample size in these low-quality studies and the non-RFLP subgroup, which may obscure any potential association.

An increasing number of studies have found that inflammation has strong effects on the pathogenesis of atherosclerosis, CAD, and its associated complications, such as MI, which are in turn widely thought to be correlated with inflammatory processes.^[5,31]^ IL-1β, a crucial mediator of inflammation, is secreted by macrophages, thrombocytes, and injured endothelium^[32,33]^ and plays a primary role in inflammatory reactions and atherosclerosis. Recently, many studies have demonstrated that IL-1β has pathogenic effects in many human conditions, including cardiovascular diseases.^[34,35]^ Kirii et al^[36]^ also proved that atherosclerotic lesions in ApoE^−/−^ IL-1β^−/−^ mice were effectively reduced by approximately 33% compared with lesions in ApoE^−/−^ mice, which suggests that IL-1β promots atherosclerosis.^[36]^ Moreover, inflammatory responses show a high interindividual difference and have been linked to single-nucleotide genetic polymorphisms in the IL-1β gene.^[37–39]^ A SNP at position + 3954 of the *IL-1β* gene, which involves the replacement of cytosine by thymine, leads to the emergence of a low-frequency allele that is related to the overproduction of IL-1β.^[40]^ In addition, Pociot et al^[14]^ demonstrated that IL-1β + 3954C/T was correlated with elevated IL-1β expression in monocytes in vitro. Indeed, several studies have indicated a role for IL-1β + 3954C/T in risk assessments for numerous inflammatory diseases due to increased IL-1β production.^[41,42]^ Thus, this polymorphism of the IL-1β gene (+3954C/T) may increase IL-1β expression, which could aggravate inflammation and finally increase the risk of MI.

We investigated the role of IL-1β + 3954C/T in relation to MI. No significant association between IL-1β + 3954C/T and MI was observed in a recessive model, homozygote model, or heterozygote model, which was coincident with the findings of previous studies.^[16,21,22]^ However, a significant relation between IL-1β + 3954C/T and MI was found in an allelic comparison and a dominant model. Previously, Tulyakova et al^[15]^ and Zeybek et al^[17]^ and reached the similar conclusion that the T allele of IL-1β + 3954C/T was related to an increased risk of MI. IL-1β may promote atherosclerosis development through different biological functions^[43]^ and lead to the production of several pro-inflammatory factors such as interleukin-6, fibrinogen, and C-reactive protein.^[44]^ In addition, IL-1β + 3954C/T is related to increased IL-1β production.^[14,40]^ All of the above studies support our meta-analysis findings that the T allele of IL-1β + 3954C/T significantly increases MI risk. Thus, individuals with the T allele of IL-1β + 3954C/T might have an increased susceptibility to MI due to a more severe inflammatory status. A subgroup analysis by ethnicity showed that a significant risk of MI susceptibility was observed in the allelic comparison, homozygote model, and dominant model among Caucasian populations. However, no significant results were observed in Asian populations. Some studies have revealed that IL-1β + 3954C/T is related to an increased risk of MI in Caucasian populations.^[15,17]^ However, Daraei et al^[19]^ demonstrated that the TT genotype of the IL-1β + 3954C/T polymorphism was associated with a significant MI-protective effect in an Asian population, and Wang et al^[16]^ showed that IL-1β + 3954C/T was not correlated with MI risk in a Chinese population. MI is a multifactorial disease and polymorphisms may also have different effects on populations as a result of diverse environmental factors. Thus, ethnographic heterogeneity, along with specific hereditary backgrounds and living conditions, could determine the different effects of the IL-1β + 3954C/T polymorphism.

In our meta-analysis, we utilized a much larger total sample size than did previous studies to evaluate the effect of the IL-1β + 3954C/T polymorphism in MI. In addition, heterogeneity was not found in the pooled results. Thus, our results are more reliable than those of previous studies. However, our meta-analysis has some limitations. First, MI has a multifactorial condition and several factors were not clear in the included studies, such as smoking, living habits, and serum lipid levels. Therefore, we cannot properly assess the association between IL-1β + 3954C/T and MI in relation to these factors. Second, although we performed a systematic search to access as much of the relevant literature as possible, it is possible that we missed some studies. Finally, only 2 studies involving in Asian populations were included and they deviated from Hardy–Weinberg disequilibrium, which may have led to unreliable results for these Asian populations. Thus, more in-depth studies with large sample sizes are required to evaluate these association in Asian populations.

In conclusion, our meta-analysis proved that IL-1β + 3954C/T is associated with MI susceptibility, especially among Caucasian populations.

## Acknowledgment

We are indebted to Huayue Lin, who helped with our study.

## Author contributions

**Conceptualization:** Yizhen Fang, Huabin Xie, Zhiyuan Lin.

**Data curation:** Yizhen Fang, Huabin Xie, Zhiyuan Lin.

**Formal analysis:** Yizhen Fang, Huabin Xie, Zhiyuan Lin.

**Investigation:** Yizhen Fang, Huabin Xie, Zhiyuan Lin.

**Methodology:** Yizhen Fang, Huabin Xie, Zhiyuan Lin.

**Software:** Yizhen Fang, Huabin Xie, Zhiyuan Lin.

**Supervision:** Huabin Xie.

**Validation:** Yizhen Fang, Huabin Xie, Zhiyuan Lin.

**Writing – original draft:** Yizhen Fang.
